# The role of obesity-related cardiovascular remodelling in mediating incident cardiovascular outcomes: a population-based observational study

**DOI:** 10.1093/ehjci/jeac270

**Published:** 2023-01-20

**Authors:** Liliana Szabo, Celeste McCracken, Jackie Cooper, Oliver J Rider, Hajnalka Vago, Bela Merkely, Nicholas C Harvey, Stefan Neubauer, Steffen E Petersen, Zahra Raisi-Estabragh

**Affiliations:** NIHR Barts Biomedical Research Centre, William Harvey Research Institute, Queen Mary University of London, Charterhouse Square, London EC1M 6BQ, UK; Barts Heart Centre, St Bartholomew’s Hospital, Barts Health NHS Trust, West Smithfield, London EC1A 7BE, UK; Heart and Vascular Center, Semmelweis University, 1122, Budapest, Varosmajor utca 68, Hungary; Division of Cardiovascular Medicine, Radcliffe Department of Medicine, University of Oxford, National Institute for Health Research Oxford Biomedical Research Centre, Oxford University Hospitals NHS Foundation Trust, Oxford OX3 9DU, UK; NIHR Barts Biomedical Research Centre, William Harvey Research Institute, Queen Mary University of London, Charterhouse Square, London EC1M 6BQ, UK; Division of Cardiovascular Medicine, Radcliffe Department of Medicine, University of Oxford, National Institute for Health Research Oxford Biomedical Research Centre, Oxford University Hospitals NHS Foundation Trust, Oxford OX3 9DU, UK; Heart and Vascular Center, Semmelweis University, 1122, Budapest, Varosmajor utca 68, Hungary; Heart and Vascular Center, Semmelweis University, 1122, Budapest, Varosmajor utca 68, Hungary; MRC Lifecourse Epidemiology Centre, University of Southampton, Southampton General Hospital, Tremona Road, Southampton SO16 6YD, UK; NIHR Southampton Biomedical Research Centre, University of Southampton and University Hospital Southampton NHS Foundation Trust, Tremona Road, Southampton SO16 6YD, UK; Division of Cardiovascular Medicine, Radcliffe Department of Medicine, University of Oxford, National Institute for Health Research Oxford Biomedical Research Centre, Oxford University Hospitals NHS Foundation Trust, Oxford OX3 9DU, UK; NIHR Barts Biomedical Research Centre, William Harvey Research Institute, Queen Mary University of London, Charterhouse Square, London EC1M 6BQ, UK; Barts Heart Centre, St Bartholomew’s Hospital, Barts Health NHS Trust, West Smithfield, London EC1A 7BE, UK; Health Data Research UK, Gibbs Building, 215 Euston Rd, London NW1 2BE, UK; Alan Turing Institute, British Library, 96 Euston Rd, London NW1 2DB, UK; NIHR Barts Biomedical Research Centre, William Harvey Research Institute, Queen Mary University of London, Charterhouse Square, London EC1M 6BQ, UK; Barts Heart Centre, St Bartholomew’s Hospital, Barts Health NHS Trust, West Smithfield, London EC1A 7BE, UK

**Keywords:** obesity, body mass index, waist-to-hip ratio, cardiac magnetic resonance imaging, cardiovascular remodelling, incident cardiovascular outcomes, disease mechanisms, mediation

## Abstract

**Aims:**

We examined associations of obesity with incident cardiovascular outcomes and cardiovascular magnetic resonance (CMR) phenotypes, integrating information from body mass index (BMI) and waist-to-hip ratio (WHR). Then, we used multiple mediation to define the role of obesity-related cardiac remodelling in driving obesity-outcome associations, independent of cardiometabolic diseases.

**Methods and results:**

In 491 606 UK Biobank participants, using Cox proportional hazard models, greater obesity (higher WHR, higher BMI) was linked to significantly greater risk of incident ischaemic heart disease, atrial fibrillation (AF), heart failure (HF), all-cause mortality, and cardiovascular disease (CVD) mortality. In combined stratification by BMI and WHR thresholds, elevated WHR was associated with greater risk of adverse outcomes at any BMI level. Individuals with overweight BMI but normal WHR had weaker disease associations. In the subset of participants with CMR (*n* = 31 107), using linear regression, greater obesity was associated with higher left ventricular (LV) mass, greater LV concentricity, poorer LV systolic function, lower myocardial native T1, larger left atrial (LA) volumes, poorer LA function, and lower aortic distensibility. Of note, higher BMI was linked to higher, whilst greater WHR was linked to lower LV end-diastolic volume (LVEDV). In Cox models, greater LVEDV and LV mass (LVM) were linked to increased risk of CVD, most importantly HF and an increased LA maximal volume was the key predictive measure of new-onset AF. In multiple mediation analyses, hypertension and adverse LV remodelling (higher LVM, greater concentricity) were major independent mediators of the obesity–outcome associations. Atrial remodelling and native T1 were additional mediators in the associations of obesity with AF and HF, respectively.

**Conclusions:**

We demonstrate associations of obesity with adverse cardiovascular phenotypes and their significant independent role in mediating obesity–outcome relationships. In addition, our findings support the integrated use of BMI and WHR to evaluate obesity-related cardiovascular risk.

## Introduction

The obesity pandemic is a global public health priority and represents a major risk factor for cardiovascular disease (CVD) and premature mortality.^1^ Obesity is traditionally defined using body mass index (BMI), a correlate of subcutaneous adiposity. However, growing evidence supports a more heterogeneous nature of obesity phenotype, incorporating differential patterns of regional body fat distribution, broadly comprising subcutaneous and visceral adiposity. Visceral fat tissue is located around solid organs and has distinct metabolic features.^2^ Waist-to-hip ratio (WHR) is a simple measure of central obesity that approximates body shape and correlates with abdominal visceral adiposity.^3^ Expert panels increasingly recommend the integration of BMI and WHR for the characterization of obesity.^4,5^ However, few studies have assessed the utility of incorporating both measures for evaluating the relationships of obesity with cardiovascular outcomes in large population cohorts.

Despite widespread recognition of obesity as a major risk factor for CVD, the mechanisms through which it promotes disease are incompletely understood. A large proportion of adverse cardiovascular associations of obesity are attributed to obesity as a driver of cardiometabolic diseases, such as hypertension and diabetes.^1^ However, obesity may also impact cardiovascular health through other independent biological pathways.^1^ For example, myocardial accumulation of triglycerides and their products have been linked to direct cardiac lipotoxicity.^6^ This and many other direct and indirect pathways may lead to myocardial disarray, dysfunction, and fibrosis.^2,7^ The role of obesity-related cardiovascular remodelling in driving associated cardiovascular risk has not been previously examined.

Cardiovascular imaging phenotypes reflect organ-level remodelling in response to a wide range of exposures and provide reliable indicators of cardiovascular health.^8^ Evaluating the relationships between obesity and cardiovascular imaging phenotypes may provide novel insights into the mechanisms driving the adverse relationships between obesity and cardiovascular outcomes. Previous studies demonstrate unhealthy cardiac remodelling patterns in obesity,^7^ using echocardiography worse diastolic function and adverse deformation patterns have also been described.^9,10^ However, cohort studies using cardiovascular magnetic resonance (CMR) imaging are limited to simplistic volumetric indices.^11–14^

In this study of the UK Biobank cohort, we evaluate the role of obesity-related cardiovascular remodelling in mediating incident cardiovascular outcomes. First, we examine the links between obesity and incident cardiovascular events, integrating information from BMI and WHR. Second, we examine associations of obesity with CMR measures of cardiac structure, function, and myocardial tissue composition. Finally, we use multiple mediation analysis to quantify the role of obesity-related cardiovascular remodelling in driving associations with incident outcomes, independent of cardiometabolic diseases.

## Methods

### Study population

The UK Biobank is a cohort study including more than 500 000 individuals from across the UK. Participants aged 40–69 years old were identified through National Health Service (NHS). The baseline assessment incorporated socio-demographics, lifestyle, environmental factors, medical history, and physical measures described in the study protocol.^15^ Incident health outcomes are prospectively tracked through linkages with national electronic health records, including hospital episode statistics (HES) and death registers. The UK Biobank Imaging Study aims to scan a randomly selected 20% (*n* = 100 000) subset of the original UK Biobank participants. The pre-defined imaging protocol consists of multiorgan multimodality imaging, including CMR.^16^

### Measures of obesity

Body size measures were performed as part of the UK Biobank assessments using standardized protocols and equipment. Height was measured using the Seca 202 height measure (Seca, Germany). Waist (natural indent) and hip (widest point) circumferences were measured over light clothes with the Seca-200 tape measure. Weight measures were taken using the Tanita BC418MA body composition analyser (Tanita, Japan).

We calculated BMI by dividing weight in kilograms by height in metres squared. We calculated WHR by dividing waist circumference by hip circumference. We considered BMI and WHR as both continuous and categorical variables. BMI categories were as follows: (i) normal BMI 18.5–24.9 kg/m^2^, (ii) overweight BMI 25–29.9 kg/m^2^, and (iii) obese BMI >30 kg/m^2^. For WHR, we used abdominal obesity cut-off points of 0.85 for women and 0.90 for men.

### Ascertainment of outcomes

The following incident CVDs were considered: any CVD, ischaemic heart disease (IHD), heart failure (HF), and atrial fibrillation (AF). We also included all-cause mortality and CVD mortality (any CVD recorded as the primary cause of death). Outcomes were extracted using record linkage to HES and death register data with diseases recorded according to International Classification of Disease (ICD) codes (see Supplementary material online, *Table S1*).

### CMR measures

Native CMR scans were performed according to a pre-defined acquisition protocol^17^ using 1.5 Tesla scanners (MAGNETOM Aera, Syngo Platform VD13A, Siemens Healthcare). The protocol included standard long-axis images and a short-axis stack covering both ventricles from base to apex, all acquired using balanced steady-state free precession sequences. CMR images were analysed using a fully automated quality-controlled pipeline.^18^ We included the following measures of left ventricular (LV) and left atrial (LA) structure and function: LV end-diastolic volume (LVEDV), mass (LVM), concentricity index (LVM:LVEDV), ejection fraction (LVEF), LA maximal volume (LAV), and LA ejection fraction (LAEF). We included LV global function index (LVGFI) as an additional measure of LV function, defined as LVSV/LV global volume × 100, where LV global volume was calculated as the sum of the LV mean cavity volume [(LV end-diastolic volume + LV end-systolic volume)/2] and myocardium volume (LV mass/density). The density of LV was specified as 1.05 g/mL. LVGFI is a measure of LV function that incorporates ventricular structure and has been shown to have more reliable associations with disease in population cohorts compared to LVEF.^8,19,20^

The CMR protocol also included myocardial native T1 mapping sequence^21^ in one midventricular short-axis slice. Global myocardial native T1 was calculated from the entire short-axis slice using a fully automated quality-controlled analysis tool.^22^ We include global native T1 in our analysis, as a measure of myocardial tissue character. The pre-specified UK Biobank protocol does not contain contrast administration.

We also considered aortic distensibility (AoD) and arterial stiffness index (ASI), as measures of arterial health.^23^ AoD provides an estimate of aortic compliance and is an indicator of local aortic bio-elastic function. We derived AoD measures from transverse cine images of the thoracic aorta using a previously validated automated quality controlled tool. ASI is an indicator of large artery stiffness derived from a pulse waveform contour obtained from finger plethysmography.

### Definition of covariates

**Figure 1:**
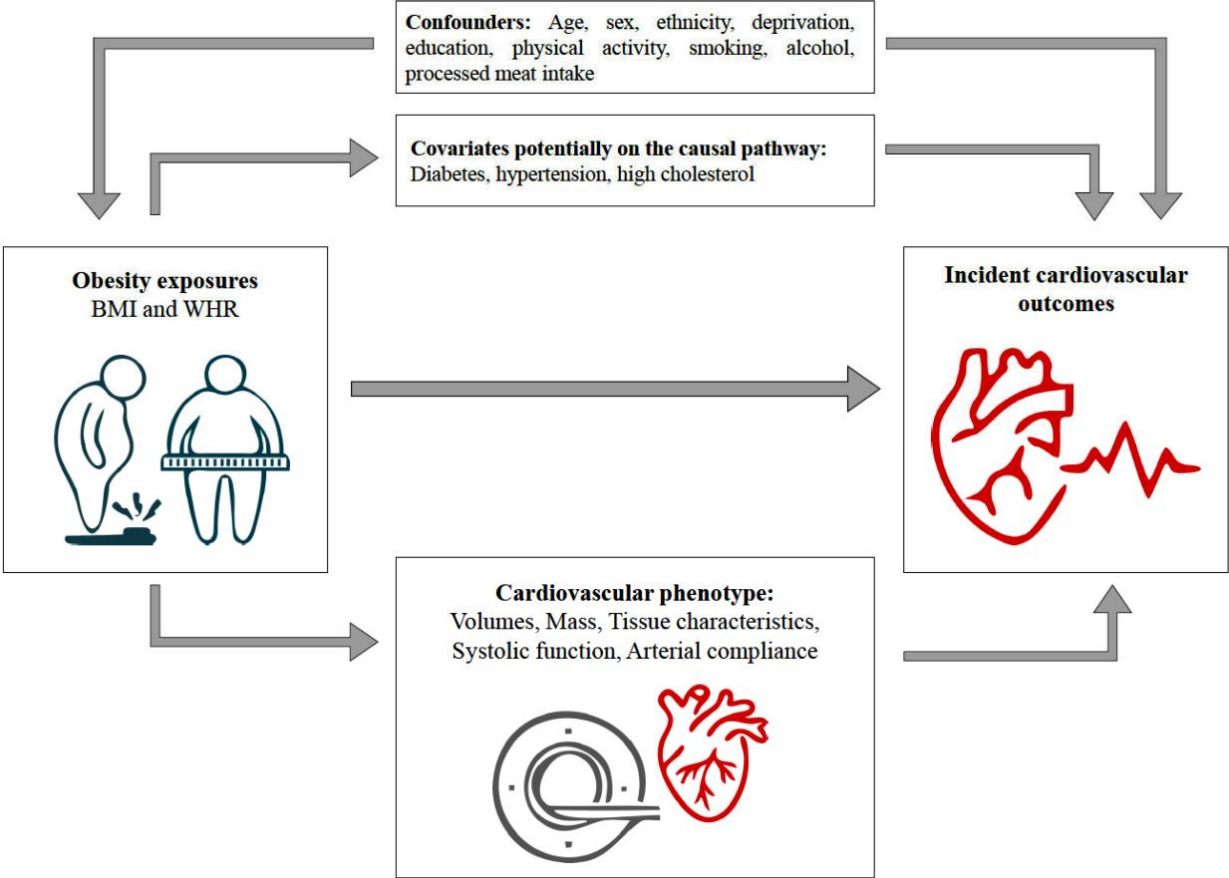
Study flowchart. Covariates considered in the relationship between obesity exposure and incident cardiovascular outcomes. BMI, body mass index; WHR, wait-to-hip ratio.

Covariates were selected based on biological plausibility and reported associations with obesity and incident cardiovascular outcomes in existing literature (*Figure 1*, Supplementary material online, *Table S2*). We adjusted for potential confounders (age, sex, ethnicity, material deprivation, education, smoking, alcohol intake, poor dietary practises, and physical activity) to estimate the magnitude of the exposure–outcome associations. We also identified the following cardiometabolic diseases as potentially lying on the causal pathway: diabetes, hypertension, and hypercholesterolaemia. We used age and sex as recorded at baseline.

### Statistical analysis

Statistical analysis was performed using R version 4.1.0. and RStudio. For associations with incident outcomes, we used the baseline set with obesity exposures (BMI and WHR) defined at baseline recruitment and incident outcomes tracked thereafter; this allowed the inclusion of a large sample with sufficient long-term follow-up^15^ (*Figure 1*). We used Cox proportional hazard regression to estimate the associations of BMI and WHR exposures (as continuous metrics) with incident CVDs (IHD, AF, HF, and any CVD) and mortality outcomes (all cause, CVD).^24^ The results are reported as hazard ratios (HRs), per 1 standard deviation (SD) increase in BMI or WHR, and 95% confidence intervals (CIs). We created hierarchical models with incremental inclusion of covariates to understand their influence on the main obesity–outcome associations. Model 1 was adjusted by age and sex; Model 2 was adjusted by Model 1 variables plus ethnicity, Townsend deprivation score, education, physical activity, alcohol consumption frequency, smoking, and processed meat intake (i.e. true confounders). Our fully adjusted model, Model 3, was adjusted by Model 2 variables plus diabetes, hypertension, and high cholesterol.

To understand potential interactions between obesity subtypes and the usefulness of their integration in clinical assessments, we created obesity strata based on WHO cut-offs for BMI and WHR, as outlined previously. We stratified the sample based on both BMI and WHR expressed as categorical exposures, creating six obesity subtypes. Individuals with normal BMI and normal WHR were considered the reference level. We report HR and 95% CI related to each obesity category against the reference level, adjusting for confounders as before.

In participants with CMR data available, we used multivariable linear regression to estimate the associations of BMI and WHR (as continuous variables) with selected cardiovascular phenotypes. To allow comparison of the magnitude of effects across CMR metrics, we report standardized beta-coefficients and raw unit coefficients with corresponding 95% CIs and *P*-values. As the main exposures (BMI and WHR) of our study are highly correlated with body size measures, we did not scale CMR metrics in our models to such measures (body surface area or height square).

Finally, we explored the role of cardiometabolic disease and CMR measures in mediating the relationships between obesity and incident outcomes. Towards this, we first describe the links between CMR measures and incident CVD outcomes, using Cox regression models. Next, we applied the Multiple Mediation Analysis for Big Data Sets package (mmabig^25^) to quantify the proportion of obesity–outcome effects mediated through cardiac remodelling (CMR metrics), hypertension, diabetes, and high cholesterol. CIs for coefficients in the mediation models were estimated across 500 bootstrapped replicates.

*P*-values were for two-tailed tests and were adjusted for multiple testing using a false discovery rate of 5% across exposure variables, giving an overall cut-off of 0.01.

## Results

### Description of baseline characteristics

The baseline dataset was available for 491 606 participants (see Supplementary material online, *Table S3* and *Figure S1*). The mean age was 56.5 (±8.1) years and the sample included 54.3% women. A total of 24.5% of participants had BMI in the obese range (>30 kg/m^2^), and almost half (49.2%) had elevated WHR. A notable proportion of participants with normal BMI had elevated WHR (21.5% in the baseline set), rising to 55.7% in the overweight BMI group at and a majority 74.8% in the obese BMI group. CMR data were available for 31 107 participants. Participant characteristics in the baseline and CMR set were broadly comparable, although the imaging set was healthier with fewer vascular risk factors and prevalent CVDs.

### Incident events

The censor date was 26 March 2021 for mortality data and HES outcomes, giving an average follow-up of 12.2(±0.9) years for the baseline set and 3.8(±1.3) years for the imaging. Within the baseline set, we observed the incidence of any CVDs in 10.4% of participants, with the most common incident CVD being IHD occurring in 6.4% of participants. Within the baseline set, 6.8% of participants died, and 12% of deaths were primarily attributed to CVDs. Within the CMR set, 1.2% of participants died, with 11.5% of deaths due primarily to CVD causes (see Supplementary material online, *Table S1*).

### Associations between obesity and incident cardiovascular events

In fully adjusted Cox regression models, higher BMI and WHR were associated with a significantly higher risk of all outcomes considered (*Table 1*). In fully adjusted models, for every SD (4.4 kg/m^2^) increase in BMI, there was 34% greater risk of incident HF, 27% greater risk of incident AF, 18% greater risk of incident IHD, 5% greater risk of all-cause mortality, and 23% greater risk of CVD mortality. Similarly, every SD increase in WHR (0.09) was associated with 33% greater risk of incident HF, 18% greater risk of incident AF, 24% greater risk of incident IHD, 19% greater risk of all-cause, and 32% greater risk of CVD mortality.

**Table 1:** Associations of obesity metrics (as continuous variables) with incident CVDs and mortality outcomes

Incident disease	BMI (log)			WHR		
	Model 1	Model 2	Model 3	Model 1	Model 2	Model 3
Any CVD	1.33*	1.30*	1.22*	1.38*	1.33*	1.22*
	[1.32, 1.34]	[1.29, 1.32]	[1.20, 1.23]	[1.37, 1.40]	[1.31, 1.34]	[1.20, 1.23]
	<1.0 × 10^−300^	<1.0 × 10^−300^	<1.0 × 10^−300^	<1.0 × 10^−300^	<1.0 × 10^−300^	7.05 × 10^−225^
Ischaemic heart disease	1.33*	1.29*	1.18*	1.46*	1.38*	1.24*
	[1.31, 1.34]	[1.27, 1.30]	[1.16, 1.19]	[1.44, 1.48]	[1.36, 1.40]	[1.22, 1.26]
	<1.0 × 10^−300^	<1.0 × 10^−300^	1.89 × 10^−155^	<1.0 × 10^−300^	<1.0 × 10^−300^	3.17 × 10^−166^
Atrial fibrillation	1.36*	1.35*	1.27*	1.30*	1.27*	1.18*
	[1.35, 1.38]	[1.33, 1.37]	[1.25, 1.28]	[1.28, 1.32]	[1.25, 1.29]	[1.16, 1.20]
	<1.0 × 10^−300^	<1.0 × 10^−300^	3.11 × 10^−281^	8.66 × 10^−239^	2.85 × 10^−190^	1.04 × 10^−80^
Heart failure	1.59*	1.52*	1.34*	1.67*	1.54*	1.33*
	[1.57, 1.62]	[1.49, 1.54]	[1.32, 1.37]	[1.64, 1.71]	[1.51, 1.57]	[1.30, 1.36]
	<1.0 × 10^−300^	<1.0 × 10^−300^	3.43 × 10^−237^	<1.0 × 10^−300^	<1.0 × 10^−300^	7.38 × 10^−132^
All-cause mortality	1.17*	1.12*	1.05*	1.39*	1.28*	1.19*
	[1.16, 1.18]	[1.11, 1.14]	[1.03, 1.06]	[1.37, 1.41]	[1.27, 1.30]	[1.18, 1.21]
	1.51 × 10^−174^	4.74 × 10^−91^	1.49 × 10^−13^	<1.0 × 10^−300^	1.15 × 10–256	1.76 × 10^−119^
CVD mortality	1.56*	1.46*	1.23*	1.80*	1.61*	1.32*
	[1.51, 1.61]	[1.42, 1.51]	[1.19, 1.27]	[1.73, 1.87]	[1.55, 1.68]	[1.26, 1.38]
	5.19 × 10^−176^	2.38 × 10^−127^	3.38 × 10^−34^	1.09 × 10^−199^	2.17 × 10^−121^	1.74 × 10^−37^

Results are HRs associated with 1 SD increase in obesity [BMI (log) or WHR], 95% CIs, and *P*-values from Cox models relating the exposures to incident disease/events in the full sample. One SD BMI (log) = 4.4 kg/m^2^ and 1 SD WHR = 0.09. *Represent significant associations.

We observed a significantly larger magnitude of effect with WHR compared with BMI in the associations with incident IHD (HR 1.46 vs. 1.33) and with both CVD mortality (HR 1.80 vs. 1.56) and all-cause mortality (HR 1.39 vs. 1.17). BMI showed a larger effect size (but with overlap of CIs) in the association with incident AF.

**Figure 2:**
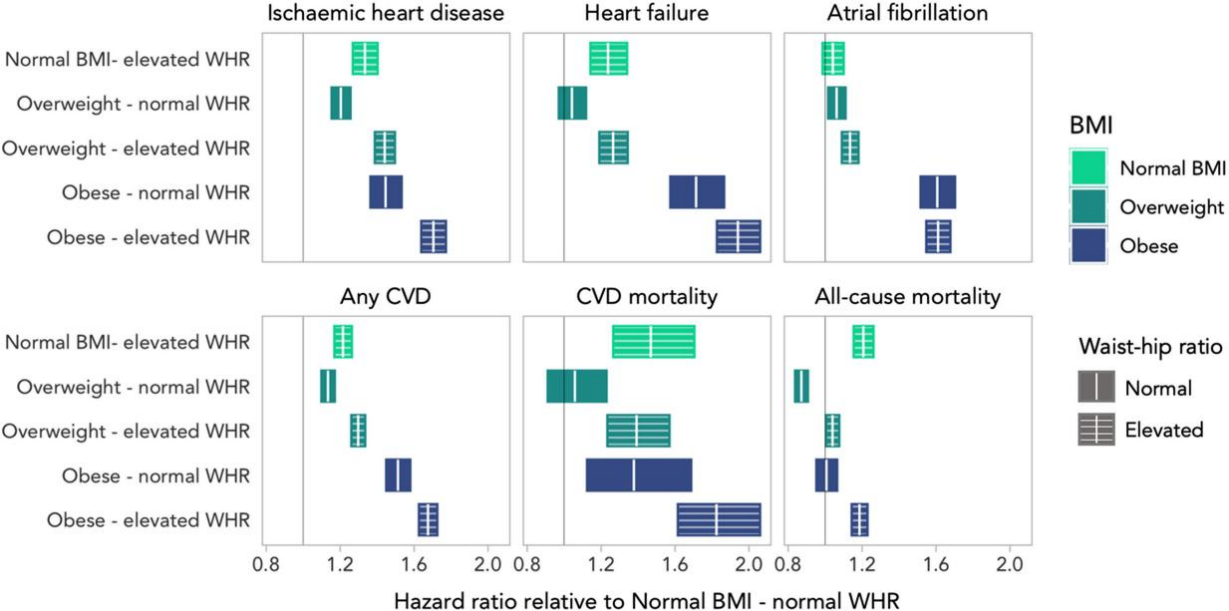
Results from Cox proportional hazards models relating obesity category to incident disease in the full sample. Each panel represents one fully adjusted model, where the baseline category is normal BMI–normal WHR. Rectangles indicate the 95% CI for the HR.

In the combined stratification by both WHR and BMI (see Supplementary material online, *Table S4*, *Figure 2*), elevated WHR was linked to adverse outcomes in any BMI combination. Individuals with ‘normal BMI-elevated WHR’ had a significantly higher risk of incident outcomes (all significant except AF) compared with those with ‘normal BMI-normal WHR’ (*Figure 2*). Individuals in the ‘overweight-normal WHR’ category had significant but weaker associations with disease than their ‘elevated WHR’ counterparts, and a significantly lower risk of overall death. For individuals in the ‘obese-elevated WHR’ group, adverse outcome associations were augmented compared with when each exposure was assessed alone (in both categorical and continuous approaches).

### Association of obesity exposure with CMR metrics

**Figure 3:**
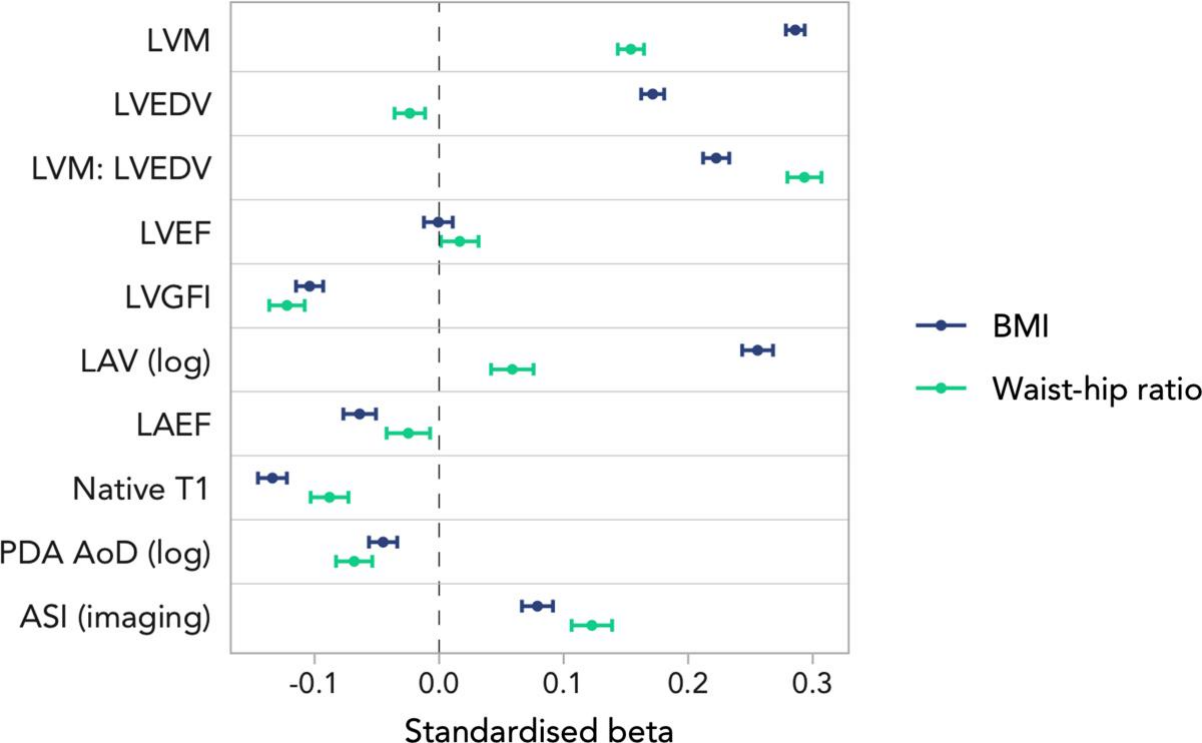
Linear regression results for raw CMR metrics displaying beta coefficients and 95% CIs per 1SD increase in (log)BMI or WHR in the imaging subset.

We summarize the mean CMR metrics for the whole imaging set and in combined stratification by both WHR and BMI obesity cut-offs (see Supplementary material online, *Table S5*). In fully adjusted models, greater obesity (both BMI and WHR) was associated with an adverse pattern of cardiovascular remodelling across all metrics considered (*Figure 3*). Specifically, greater obesity was linked to higher LVM, a more concentric pattern of LV hypertrophy (higher LVM: LVEDV), and poorer LV function (lower LVGFI). Higher BMI was linked to higher, whilst greater WHR was linked to lower LVEDV. Higher obesity metrics were linked to significantly lower myocardial native T1. With regards to LA remodelling, greater obesity was linked to larger LA volumes (higher LAV), and poorer LA function (lower LAEF). Higher BMI and WHR were also linked to lower (unhealthy) arterial compliance by both AoD and ASI.

While the direction of associations was broadly consistent between the two obesity metrics, we observed a larger magnitude of effect with BMI compared with WHR in the associations with LVM, native T1, LAV, and LAEF. Whilst WHR showed stronger associations with LVM:LVEDV and ASI. BMI and WHR showed similar strength of effect with LVGFI (*Figure 3*).

### The association of CMR metrics with incident outcomes

Towards considering the mediation of obesity–outcome associations through cardiac alterations, we tested the association of selected CMR metrics with our outcomes of interest. In fully adjusted Cox regression models, larger LVEDV, higher LVM, and poorer LV function by both LVEF and LVGFI were associated with incident CVD (see Supplementary material online, *Table S6).* These relationships appeared most prominent for HF. Higher native T1 relaxation times were linked to greater incident AF, HF, CV mortality, and all-cause mortality. Higher LVM:LVEDV was associated with any CVD and IHD. Not surprisingly, the relationships with LA metrics were strongest in case of AF. ASI showed no association with any of the considered incident outcomes.

### Mediation analysis

**Figure 4:**
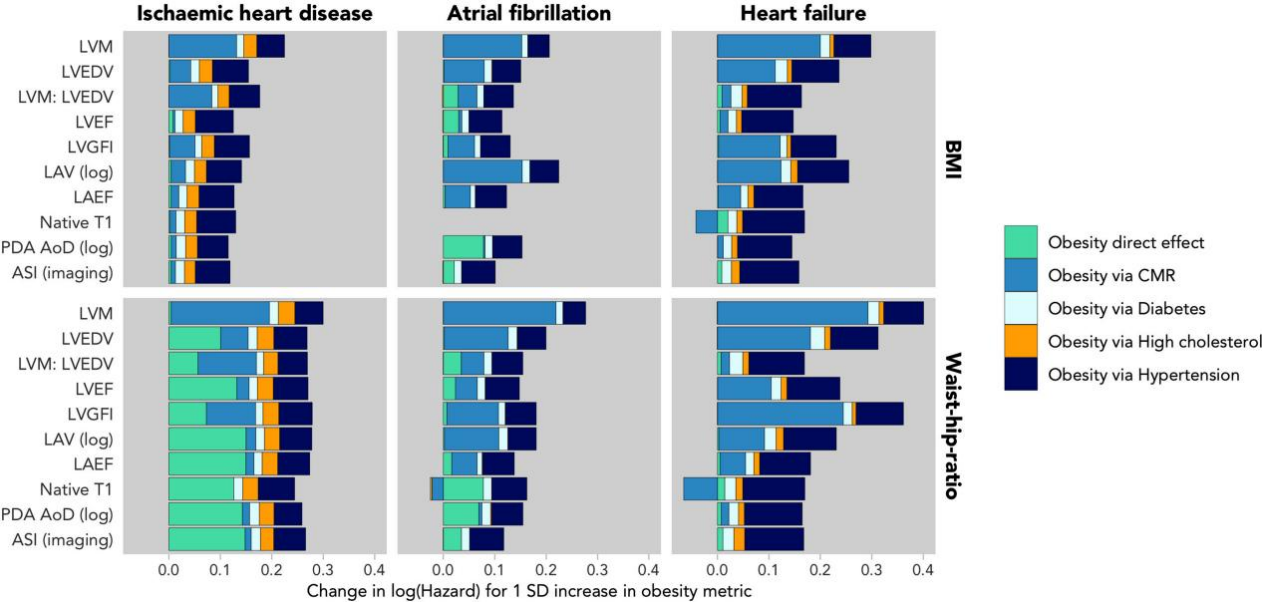
Results from fully adjusted multiple mediation models between obesity and incident CVD, mediated by the one raw CMR metric and the three cardiometabolic conditions in each iteration. The overall size of the bars corresponds to the strength of the total effect between obesity and incident disease, measured by (log)BMI on the top row and WHR on the bottom row. Each separate area of the columns reflect the size of the mediated effects via each mediator.

We used multiple mediation analyses to investigate the potential mechanisms driving the obesity–outcome associations, considering the mediation through cardiometabolic diseases (diabetes, high cholesterol, and hypertension) and CMR alterations. The mediation analysis considered three key outcomes of incident IHD, AF, and HF. Granular results are available in Supplementary material online, *Tables S7* and *S8*, and a visual summary is shown in *Figure 4*. Overall, we found that the effects of both BMI and WHR on incident outcomes were potentially mediated by obesity-induced adverse cardiac remodelling and hypertension, with a smaller mediating effect of diabetes.

Adverse alterations of LV structure and function (high LVM, higher LV concentricity, and lower LVGFI) appeared as potential mediators for all three incident CVDs, potentially contributing a large proportion of the mediated effect, independent of cardiometabolic diseases. Atrial remodelling (larger LAV) was identified as an additional independent mediator of the relationship between obesity and AF. Native T1 mediated a significant fraction of the relationship between obesity and incident HF (BMI: −36%, WHR: −77%). This relationship is negative, meaning that while BMI and WHR are associated with a decreasing T1, increasing T1 value is linked to incident HF.

## Discussion

### Summary of findings

In this large population-based cohort, we consider the role of obesity-related cardiovascular remodelling in potentially driving key incident cardiovascular outcomes. We first demonstrate independent associations of BMI and WHR as independent predictors of incident outcomes. In combined stratification by both WHR and BMI, individuals with elevated WHR had a greater risk of all outcomes at any BMI group. Second, greater BMI and WHR were linked to adverse LV structure, poorer LV function, lower myocardial native T1, large LA size, poorer LA function, and lower arterial compliance. Third, we showed that greater LVM is linked to an increased risk of all CVDs considered in our study, moreover, greater LVEDV and LAV are linked to an increased risk of HF and AF. Finally, we found that hypertension and adverse LV remodelling were the two strongest mediating factors between excess weight and incident IHD, AF, and HF using multiple mediation analyses.

### Comparison with existing research

Previous researchers have demonstrated associations of obesity with adverse CVD and mortality outcomes.^26^ Our work extends these observations through integrated modelling of BMI with WHR in a population-based cohort and by considering a wider range of CVD outcomes. We demonstrate that an elevated WHR at any BMI level is linked to adverse incident CVD and mortality outcomes. Furthermore, our findings suggest that WHR may provide better discrimination of ‘pathological’ obesity than BMI alone. In particular, we observed inconsistent outcome associations for individuals classified as overweight per BMI but who had a normal WHR. These individuals had weaker associations with incident CVDs, non-significant association with HF and CVD death, and lower risk of all-cause death. Thus, it is likely that some individuals in this category were healthy, contrary to the BMI classification of overweight. In comparison, those with elevated WHR but normal BMI had elevated risk of all-cause death, CVD death, and all incident CVDs (non-significant for AF).

The paradoxical associations between mortality and overweight BMI have been previously reported.^27^ A growing body of evidence supports methodological explanations rather than a true mechanistic relationship, such as misclassification bias caused by using BMI as a sole measure of obesity or unmeasured confounding.^2^ Our findings support the characterization of obesity with integration of BMI and WHR to better distinguish health from disease (minimize misclassifications) and to strengthen cardiovascular outcome prediction.

Previous studies have reported the associations of greater obesity with adverse cardiac remodelling across a limited range of phenotypes. In a study of 5098 participants of the MESA cohort, Turkbey *et al.*^28^ demonstrate, consistent with our observations, association of greater obesity with greater LVM and concentric LV remodelling without change in LVEF. In an echocardiography study of 4343 participants of the ARIC study, Bello *et al.*^29^ also observed the association of greater obesity with greater LVM. Our findings support these existing reports in a much larger cohort and using the reference standard modality of CMR. A novel finding of our research is that higher BMI was linked to higher, whilst greater WHR was linked to lower LVEDV. Clearly showing the disparity of the effect of BMI and WHR on remodelling. Possible explanations for this observation include higher cardiac output as opposed to increased load in the driving mechanisms of obesity-related cardiac remodelling, as well as an increased arterial stiffness (causing increased afterload) and/or metabolic dysregulation. Furthermore, whilst we also observed non-significant associations of obesity with LVEF (as per Turkbey *et al.*^28^), we demonstrate associations of greater obesity with poorer LV function by lower LVGFI, an emerging marker of LV systolic function.^30^

We are the first to report associations of obesity with myocardial native T1 in a large cohort. This metric provides a non-invasive indicator of myocardial tissue character. In general, higher native T1 is linked to a greater myocardial fibrosis and adverse outcomes. Adipose tissue shortens T1 relaxation time.^31^ We demonstrate association of greater obesity with lower native T1; however, higher native T1 was found to be linked to greater risk of incident HF. A possible interpretation is that these associations indicate different stages of obesity-related cardiac disease. The associations of greater obesity with lower native T1 observed in the whole sample may indicate subclinical myocardial lipid accumulation as a feature of early-stage obesity remodelling. Whilst in later stages, fibrotic degeneration replaces lipid accumulation leading to clinical disease and pump failure, hence the association of higher native T1 with incident HF. Previous studies have made the link between myocardial steatosis and poorer cardiac health^32^ and between myocardial fibrosis and adverse cardiovascular outcomes.^33^ Notably, native T1 may also be elevated by other potentially pathologic processes, such as subacute inflammation and myocardial hyper-perfusion.^31^ Such alterations may represent other biologic pathways through which obesity-mediated HF occurs. Thus, our findings present new insight into potential disease mechanisms driving the adverse cardiovascular consequences of obesity, which merit further study.

Our findings indicate an association of greater obesity with greater LA dilatation and poorer LA function. These observations likely indicate elevated LV filling pressures and diastolic dysfunction. Consistently, Al Jaroudi *et al.*^34^ also demonstrate an association of higher BMI with poorer diastolic function in an echocardiography study of 21 666 participants. In the mediation analysis, we additionally demonstrate that LA remodelling (higher LAV) is a potential mediator of the associations between obesity and incident AF. This observation supports previous reports proposing a direct mechanistic role for obesity in driving AF through the promotion of electroanatomic remodelling.^35^

Overall, our results show that from the cluster of metabolic pathologies associated with obesity, hypertension is the most important cardiometabolic disease linking adiposity exposure to adverse CVD outcomes. This is consistent with many previous studies linking obesity to a greater risk for hypertension, which in turn is widely recognized as a major risk factor for CVD occurrence.^1^ However, here, we formally illustrate the role of hypertension in mediating the adverse clinical consequences of obesity.

Although we demonstrate a probable mediating role for hypertension, a large proportion of the obesity–outcome associations are possibly attributed to CMR alterations. CMR alterations do not occur spontaneously but rather are a response to an exposure. Firstly, obesity itself may have a direct role in altering the cardiac phenotype. Second, it is possible that obesity mediates cardiovascular remodelling through variables other than the cardiometabolic factors considered in our analysis—that is obesity is acting through other indirect biological pathways. Thirdly, a proportion of the CMR-mediated effects may be related to residual confounding from incomplete capture of cardiometabolic conditions. LV structure and function metrics had conceivable roles mediating associations of obesity with all three conditions (AF, HF, and IHD). These adverse remodelling patterns are incompletely explained by known cardiometabolic factors. Our work encourages further research to identify direct and indirect biological pathways that may be driving obesity-related cardiac remodelling.

### Clinical and research implications

Our findings demonstrate potential shortcomings rooted in the oversimplification of excess weight using BMI alone. We highlight merit in the characterization of obesity using both BMI and WHR for better capture of obesity-related cardiovascular risk. Given that these measures are highly accessible, cheap, and non-invasive their integration into existing clinical pathways is likely to be feasible across a wide range of settings. Further validation of our findings and examining practicalities of integrative BMI–WHR phenotyping of obesity in routine practice are warranted.

Our work encourages research into possible direct pathways such as lipotoxicity, obstructive sleep apnea,^36^ changes in the intracellular homeostasis, circulating hormones,^37^ oxidative stress, inflammation, and fibrosis that may all contribute to the myocardial structure's deterioration,^7^ providing therapeutic targets for medical intervention and lifestyle modification.

### Limitations

Incident outcomes are based on HES data that limits us to incident diseases recorded in a hospital setting. The UK Biobank imaging study CMR protocol did not include more extensive tissue characterization sequences such as T2 or gadolinium contrast-enhanced images. A limitation of the mediation analyses is it provides a means of apportioning variance, but does not permit an inference of causal associations. Thus, whilst it is possible to speculate on causal pathways based on our findings and what is known from the pre-existing literature, we are not able to draw any definitive conclusions from the present study. Indeed, we cannot exclude residual confounding or reverse causation due to the study's observational nature. Finally, at the time of our analysis, UK Biobank’s primary care linkage was incomplete, and medication history could be ascertained only from baseline recorded self-reports that has several limitations, therefore, we did not address this area in our current analysis.

## Conclusions

The combined use of BMI and WHR to characterize obesity may provide better discrimination of ‘pathologic’ obesity and provide better estimations of cardiovascular risk than either metric alone. We demonstrate novel associations of obesity with a wide range of adverse CMR phenotypes, which along with hypertension have important independent roles in potentially mediating obesity–outcome associations. The mediated effects attributed to ‘CMR metrics’ may represent direct obesity-related damage and/or residual effect of other metabolic exposures that are associated with obesity.

## Authors contribution

L.S., C.M., Z.R.-E., and S.E.P. conceived the idea and developed the analysis plan. C.M. led the statistical analysis and provided all figures and tables for the manuscript. J.C. supervised the statistical analysis and figure creation. C.M., L.S., and Z.R.-E. have verified the underlying data. All authors had full access to all the data in the study and accept the responsibility to submit for publication. L.S. and Z.R.-E. wrote the original manuscript. L.S., O.R., H.V., B.M., S.N., N.C.H., S.E.P., and Z.R.-E contributed to the interpretation of the data. S.E.P. and Z.R.-E. jointly supervised the work. All co-authors critically reviewed the manuscript and approved the final submitted version.

## Supplementary material

Supplementary materials are available at *European Heart Journal - Cardiovascular Imaging* online.

## Ethical approval

This study complies with the Declaration of Helsinki; the work was covered by the ethical approval for UK Biobank studies from the National Health Service (NHS) National Research Ethics Service on 17 June 2011 (Ref 11/NW/0382) and extended on 18 June 2021 (Ref 21/NW/0157) with written informed consent obtained from all participants.

## Supplementary Material

jeac270_Supplementary_Data

## Data Availability

This research was conducted using the UK Biobank resource under access application 2964. UK Biobank will make the data available to all bona fide researchers for all types of health-related research that is in the public interest, without preferential or exclusive access for any persons. All researchers will be subject to the same application process and approval criteria as specified by UK Biobank. For more details on the access procedure, see the UK Biobank website: http://www.ukbiobank.ac.uk/register-apply.
