# Supplementary material for: The role of obesity-related cardiovascular remodelling in mediating incident cardiovascular outcomes: a population-based observational study
Source: Eur Heart J Cardiovasc Imaging. 2023 Jan 20;24(7):921–9. doi: 10.1093/ehjci/jeac270 (PMC10284050; doi:10.1093/ehjci/jeac270)
Supplement: jeac270_Supplementary_Data [file jeac270_supplementary_data.docx]

**Supplementary Figure 1. Sample selection**


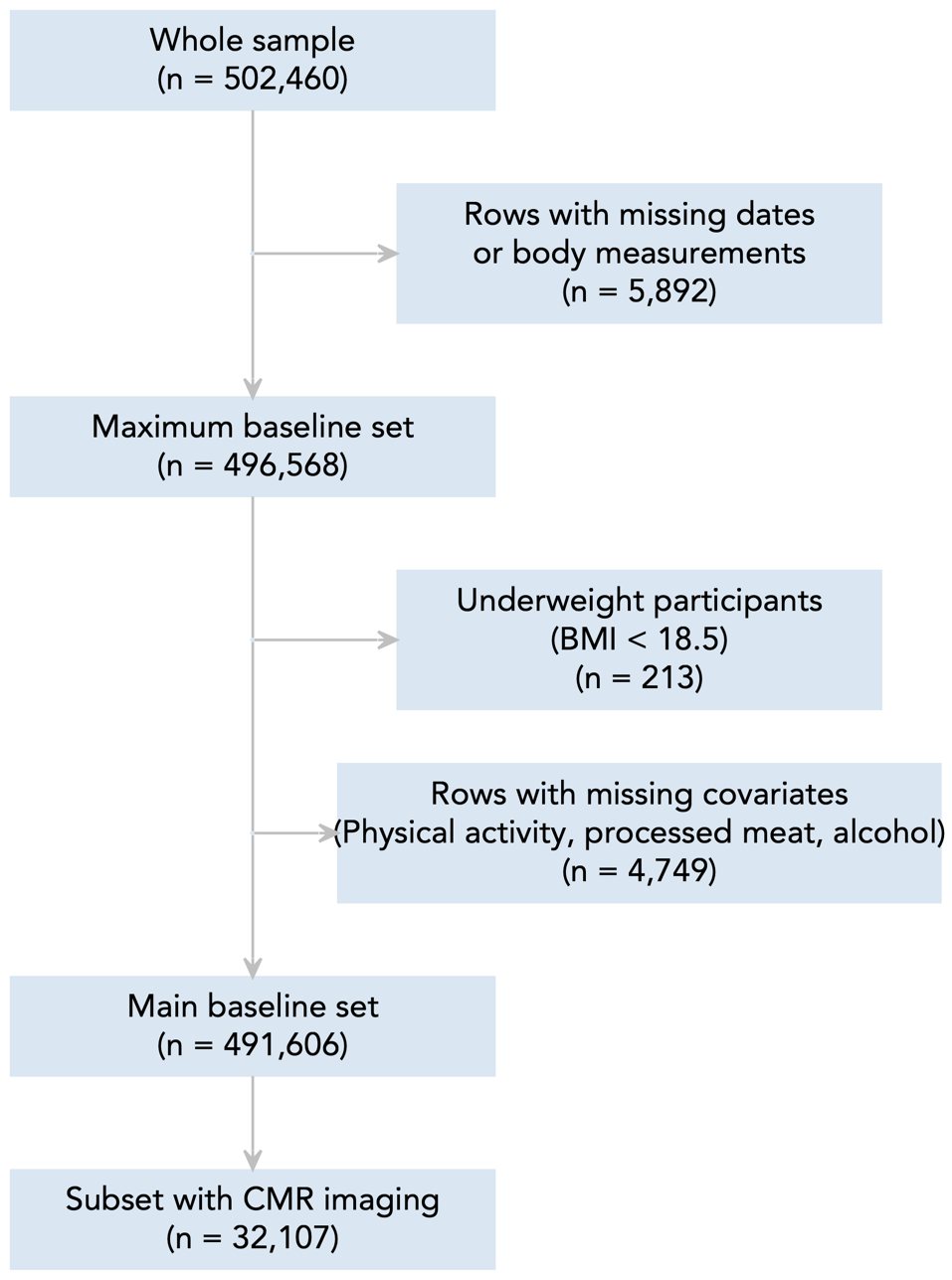


**Supplementary Figure 1 footnote.** BMI: body mass index, CMR: cardiovascular magnetic resonance.

**Supplementary Table 1. UK Biobank fields/codes for cardiovascular disease**

| **Source** | **Field ID or code** | **CVD death** | **Description** |
| --- | --- | --- | --- |
| ***Ischaemic heart disease*** | | | |
| Self-report | 20004 |  | coronary angioplasty (ptca) +/- stent |
|  | 20004 |  | coronary artery bypass grafts (cabg) |
|  | 20002 |  | heart attack/myocardial infarction |
|  | 20002 |  | heart failure/pulmonary odema |
| OPCS4 | K504 |  | K50.4 Percutaneous transluminal atherectomy of coronary artery |
|  | K503 |  | K50.3 Percutaneous transluminal injection of therapeutic substance into coronary artery NEC |
|  | K502 |  | K50.2 Percutaneous transluminal coronary thrombolysis using streptokinase |
|  | K40 |  | K40 Saphenous vein graft replacement of coronary artery |
|  | K41 |  | K41 Other autograft replacement of coronary artery |
|  | K42 |  | K42 Allograft replacement of coronary artery |
|  | K43 |  | K43 Prosthetic replacement of coronary artery |
|  | K44 |  | K44 Other replacement of coronary artery |
|  | K45 |  | K45 Connection of thoracic artery to coronary artery |
|  | K46 |  | K46 Other bypass of coronary artery |
|  | K49 |  | K49 Transluminal balloon angioplasty of coronary artery |
|  | K501 |  | K50.1 Percutaneous transluminal laser coronary angioplasty |
|  | K75 |  | K75 Percutaneous transluminal balloon angioplasty and insertion of stent into coronary artery |
| ICD9 | 410 |  | 410 Acute myocardial infarction |
|  | 411 |  | 411 Other acute and subacute forms of ischaemic heart disease |
|  | 412 |  | 412 Old myocardial infarction |
|  | 4140 |  | 4140 Coronary atherosclerosis |
|  | 4141 |  | 4141 Aneurysm of heart |
|  | 4148 |  | 4148 Other specified forms of chronic ischaemic heart disease |
|  | 4149 |  | 4149 Chronic ischaemic heart disease, unspecified |
| ICD10 | I210 | yes | I21.0 Acute transmural myocardial infarction of anterior wall |
|  | I211 | yes | I21.1 Acute transmural myocardial infarction of inferior wall |
|  | I212 |  | I21.2 Acute transmural myocardial infarction of other sites |
|  | I213 | yes | I21.3 Acute transmural myocardial infarction of unspecified site |
|  | I214 | yes | I21.4 Acute subendocardial myocardial infarction |
|  | I219 | yes | I21.9 Acute myocardial infarction, unspecified |
|  | I21X |  | I21.X Presumed acute myicardial infaction (unconfirmed) |
|  | I22 |  | I22 Subsequent myocardial infarction |
|  | I23 |  | I23 Certain current complications following acute myocardial infarction |
|  | I240 |  | I24.0 Coronary thrombosis not resulting in myocardial infarction |
|  | I241 |  | I24.1 Dressler's syndrome |
|  | I248 | yes | I24.8 Other forms of acute ischaemic heart disease |
|  | I249 | yes | I24.9 Acute ischaemic heart disease, unspecified |
|  | I251 | yes | I25.1 Atherosclerotic heart disease |
|  | I252 | yes | I25.2 Old myocardial infarction |
|  | I255 | yes | I25.5 Ischaemic cardiomyopathy |
|  | I256 | yes | I25.6 Silent myocardial ischaemia |
|  | I258 | yes | I25.8 Other forms of chronic ischaemic heart disease |
|  | I259 | yes | I25.9 Chronic ischaemic heart disease, unspecified |
| First occurrences | 131298 |  | acute myocardial infarction |
|  | 131300 |  | subsequent myocardial infarction |
|  | 131302 |  | certain current complications following acute myocardial infarction |
|  | 131304 |  | other acute ischaemic heart diseases |
| Diagnosed by doctor | 6150: 1 |  | Heart attack |
|  | 3894 |  | Age heart attack diagnosed |
| Algorithm | 42000 |  | 42000 Date of myocardial infarction |

***Supplementary Table 1 continues…***

***Supplementary Table 1 (continued)***

| **Source** | **Field ID or code** | **CVD death** | **Description** |
| --- | --- | --- | --- |
| ***Atrial fibrillation*** | | | |
| Self-report | 20002 |  | atrial fibrillation |
| ICD9 | 4273 |  | 4273 Atrial fibrillation and flutter |
| ICD10 | I480 | yes | I48.0 Paroxysmal atrial fibrillation |
| ICD10 | I481 |  | I48.1 Persistent atrial fibrillation |
| ICD10 | I482 |  | I48.2 Chronic atrial fibrillation |
| ICD10 | I489 | yes | I48.9 Atrial fibrillation and atrial flutter, unspecified |
| First occurrences | 131350 |  | Date I48 first reported (atrial fibrillation and flutter) |
| ***Heart failure*** |  |  |  |
| Self-report | 20002 |  | heart failure/pulmonary odema |
| ICD9 | 428 |  | 428 Heart failure |
| ICD9 | 4020 |  | 4020 Hypertensive heart disease, specified as malignant |
| ICD9 | 4029 |  | 4029 Hypertensive heart disease, not specified as malignant or benign |
| ICD9 | 4040 |  | 4040 Hypertensive heart and renal disease, specified as malignant |
| ICD9 | 4049 |  | 4049 Hypertensive heart and renal disease, not specified as malignant or benign |
| ICD10 | I110 | yes | I11.0 Hypertensive heart disease with (congestive) heart failure |
| ICD10 | I130 | yes | I13.0 Hypertensive heart and renal disease with (congestive) heart failure |
| ICD10 | I132 | yes | I13.2 Hypertensive heart and renal disease with both (congestive) heart failure and renal failure |
| ICD10 | I500 | yes | I50.0 Congestive heart failure |
| ICD10 | I501 | yes | I50.1 Left ventricular failure |
| ICD10 | I509 | yes | I50.9 Heart failure, unspecified |
| First occurrences | 131354 |  | Date I50 first reported (heart failure) |
| ***Additional codes included under “Any CVD”*** | | | |
| Self-report | 20002 |  | cardiomyopathy |
| Self-report | 20002 |  | hypertrophic cardiomyopathy (hcm / hocm) |
| ICD9 | 4274 |  | 4274 Ventricular fibrillation and flutter |
| ICD9 | 4275 |  | 4275 Cardiac arrest |
| ICD9 | 4255 |  | 4255 Alcoholic cardiomyopathy |
| ICD10 | I46 | yes | I46 Cardiac arrest |
| ICD10 | I472 | yes | I47.2 Ventricular tachycardia |
| ICD10 | I490 | yes | I49.0 Ventricular fibrillation and flutter |
| ICD10 | I420 | yes | I42.0 Dilated cardiomyopathy |
| ICD10 | I426 | yes | I42.6 Alcoholic cardiomyopathy |
| ICD10 | I42 |  | I42 Cardiomyopathy |
| ICD10 | I43 |  | I43 Cardiomyopathy in diseases classified elsewhere |
| ICD10 | I11 | yes | I11 Hypertensive heart disease |
| ICD10 | I13 |  | I13 Hypertensive heart and renal disease |
| First occurrences | [131346](https://biobank.ndph.ox.ac.uk/showcase/field.cgi?id=131346) |  | [Date I46 first reported (cardiac arrest)](https://biobank.ndph.ox.ac.uk/showcase/field.cgi?id=131346) |
| First occurrences | 131338 |  | cardiomyopathy |
| First occurrences | 131340 |  | cardiomyopathy in diseases classified elsewhere |
| First occurrences | 131288 |  | hypertensive heart disease |
| First occurrences | 131292 |  | hypertensive heart and renal disease |

**Supplementary Table 1 footnote**. ICD10 codes are drawn from fields 41270, 41280, 41234 and 41259; ICD9 codes are drawn from fields 41271, 41281, 41234 and 41259; OPCS4 codes are drawn from fields 41272, 41282, 41149 and 41259; Deaths codes are drawn from fields 40000, 40001 and 40023. Where a 3-digit code is given, this includes all 4-digit sub-codes, for example, I46 includes I462, I468 and I469.

**Supplementary Table 2. UK Biobank fields/codes for risk factors**

| **Source** | **Field ID or code** | **Description** |
| --- | --- | --- |
| ***Diabetes*** |  |  |
| Self-report | 20002 | Diabetes |
|  | 20002 | Type 1 diabetes |
|  | 20002 | Type 2 diabetes |
| Medications | 6153, 6177: 3 | Insulin |
| ICD9 | 250 | Diabetes mellitus |
| ICD10 | E10 | E10 Insulin-dependent diabetes mellitus |
| ICD10 | E11 | E11 Non-insulin-dependent diabetes mellitus |
| ICD10 | E12 | E12 Malnutrition-related diabetes mellitus |
| ICD10 | E13 | E13 Other specified diabetes mellitus |
| ICD10 | E14 | E14 Unspecified diabetes mellitus |
| ICD10 | O24 | O24 Diabetes mellitus in pregnancy |
| ICD10 | R73 | R73 Elevated blood glucose level |
| First occurrences | 130706 | Date E10 first reported (insulin-dependent diabetes mellitus) |
| First occurrences | 130708 | Date E11 first reported (non-insulin-dependent diabetes mellitus) |
| First occurrences | 130710 | Date E12 first reported (malnutrition-related diabetes mellitus) |
| First occurrences | 130712 | Date E13 first reported (other specified diabetes mellitus) |
| First occurrences | 130714 | Date E14 first reported (unspecified diabetes mellitus) |
| Diagnosed by doctor | 2443 | Diabetes diagnosed by doctor |
| Diagnosed by doctor | 2976 | Age diabetes diagnosed by doctor |
| Biochemistry | 30740 | Serum glucose >11.1 mmol/L |
| Biochemistry | 30750 | Glycated haemoglobin (HbA1c) > 48 |
| ***Hypertension*** |  |  |
| Self-report | 20002 | Essential hypertension |
|  | 20002 | Hypertension |
| Medications | 6153, 6177: 2 | Blood pressure medication |
| ICD10 | I10 | Essential (primary) hypertension |
| First occurrences | 131286 | Date I10 first reported (essential (primary) hypertension) |
| Diagnosed by doctor | 6150: 4 | High blood pressure |
|  | 2966 | Age high blood pressure diagnosed |
| ***High cholesterol*** |  |  |
| Self-report | 20002 | high cholesterol |
| Medications | 6153, 6177: 1 | Cholesterol lowering medication |
| ICD10 | E780 | E78.0 Pure hypercholesterolaemia |
| ICD10 | E781 | E78.1 Pure hyperglyceridaemia |
| ICD10 | E782 | E78.2 Mixed hyperlipidaemia |
| ICD10 | E783 | E78.3 Hyperchylomicronaemia |
| ICD10 | E784 | E78.4 Other hyperlipidaemia |
| ICD10 | E785 | E78.5 Hyperlipidaemia, unspecified |
| First occurrences | 130814 | Date E78 first reported (disorders of lipoprotein metabolism and other lipidaemias) |
| Biochemistry | 30690 | serum total cholesterol >7 mmol/L |

**Supplementary Table 2 footnote**:

We accessed self-reported fields for participants’ educational level, smoking status, processed meat intake, and alcohol intake. Ethnicity was categorised as White or BAME (Black, Asian and Minority Ethnic). Material deprivation is reported as the Townsend index, which measures location-based socioeconomic deprivation relative to national averages. Physical activity was measured via self-reported responses to the International Physical Activity Questionnaire (IPAQ). Participants reported their time spent in different types of exercise (walking, moderate, or vigorous), from which their overall summed metabolic equivalent (MET) minutes/week was calculated as per published guidance. Diabetes was ascertained from any of: self-report, reported use of insulin, hospital records, blood biomarkers (serum glucose > 11.1 mmol/L, or serum glycosylated haemoglobin >48 mmol/mol). Hypertension was coded based on hospital records, self-reports or self-reported use of blood pressure medication. High cholesterol was coded based on self-report, self-reported use of cholesterol-lowering medication, hospital records, or serum total cholesterol >7 mmol/L. ICD10 codes are drawn from fields 41270, 41280, 41234 and 41259; ICD9 codes are drawn from fields 41271, 41281, 41234 and 41259; Where a 3-digit code is given, this includes all 4-digit sub-codes, for example, E10 includes E100, E101 and E102 etc

**Supplementary Table 3. Participant characteristics**

| **Characteristic** | **Baseline set (n=491,606)** | **Imaging set  (n= 31,107)** |
| --- | --- | --- |
| Age | 56.5 (±8.1) | 63.3 (±7.5) |
| Female sex* | 266,997 (54.3%) | 16,519 (51.4%) |
| Ethnicity - white | 466,354 (94.9%) | 31,246 (97.3%) |
| Ethnicity - BAME | 25,252 (5.1%) | 861 (2.7%) |
| Townsend deprivation index* | -2.2 [-3.7, 0.5] | -2.7 [-3.9, -0.6] |
| Post-secondary education/qualification* | 292,232 (59.4%) | 23,384 (72.8%) |
| Smoking – never smoked | 268,208 (54.6%) | 19,809 (61.7%) |
| Smoking – previous smoker | 170,558 (34.7%) | 10,793 (33.6%) |
| Smoking – current smoker | 51,122 (10.4%) | 1,148 (3.6%) |
| Alcohol frequency – never | 38,587 (7.8%) | 2,064 (6.4%) |
| Alcohol frequency – less than once per week | 111,300 (22.6%) | 6,892 (21.5%) |
| Alcohol frequency – once or more per week | 341,719 (69.5%) | 22,891 (71.3%) |
| Processed meat frequency – less than weekly | 195,390 (39.7%) | 13,702 (42.7%) |
| Processed meat frequency – weekly | 143,778 (29.2%) | 8,745 (27.2%) |
| Processed meat frequency – two or more times/week | 152,438 (31.0%) | 9,386 (29.2%) |
| Physical activity – inactive (<600 summed METS /week) | 128,981 (26.2%) | 5,295 (16.5%) |
| Physical activity – active (600 – 2,999 summed METS /week) | 231,028 (47.0%) | 16,413 (51.1%) |
| Physical activity – very active (>3,000 summed METS/week) | 131,597 (26.8%) | 10,123 (31.5%) |
| Median BMI (kg/m^2^) | 26.8 [24.2, 29.9] | 26.0 [23.6, 28.9] |
| Normal BMI (18.5 – 24.9) | 161,106 (32.8%) | 12,815 (39.9%) |
| Overweight BMI (25 – 29.9) | 210,150 (42.7%) | 13,377 (41.7%) |
| Obese BMI (≥ 30 ) | 120,350 (24.5%) | 5,915 (18.4%) |
| Mean waist-hip ratio | 0.87 (±0.09) | 0.87 (±0.09) |
| Normal WHR | 249,758 (50.8%) | 16,344 (50.9%) |
| Elevated WHR | 241,848 (49.2%) | 15,763 (49.1%) |
| Normal BMI - normal WHR | 126,475 (25.7%) | 9,432 (29.4%) |
| Normal BMI - elevated WHR | 34,631 (7.0%) | 3,383 (10.5%) |
| Overweight - normal WHR | 92,994 (18.9%) | 5,446 (17.0%) |
| Overweight - elevated WHR | 117,156 (23.8%) | 7,931 (24.7%) |
| Obese - normal WHR | 30,289 (6.2%) | 1,466 (4.6%) |
| Obese - elevated WHR | 90,061 (18.3%) | 4,449 (13.9%) |
| Diabetes status | 30,344 (6.2%) | 1,923 (6.0%) |
| Hypertension status | 147,071 (29.9%) | 10,644 (33.2%) |
| High cholesterol status | 149,398 (30.4%) | 11,263 (35.1%) |
| Prevalent conditions |  |  |
| Any CVD | 27,168 (5.5%) | 2,327 (7.2%) |
| Ischaemic heart disease | 20,327 (4.1%) | 1,533 (4.8%) |
| Atrial fibrillation | 8,137 (1.7%) | 922 (2.9%) |
| Heart failure | 2,646 (0.5%) | 196 (0.6%) |
| Incident events |  |  |
| Any CVD | 51,077 (10.4%) | 943 (2.9%) |
| Ischaemic heart disease | 31,474 (6.4%) | 578 (1.8%) |
| Atrial fibrillation | 27,247 (5.5%) | 505 (1.6%) |
| Heart failure | 13,865 (2.8%) | 235 (0.7%) |
| All-cause mortality | 33,525 (6.8%) | 382 (1.2%) |
| CVD mortality | 4,033 (0.8%) | 44 (0.1%) |

**Supplementary Table 3 footnote.** * Sex, education and Townsend deprivation measured only at baseline. All other factors including prevalent conditions are measured at baseline for the full set, and at imaging for the imaging subset. Incident events are counted after baseline for the whole set, and after imaging for the imaging subset. Abbreviations: BAME, Black Asian and Minority ethnicities; BMI, body mass index; CVD, cardiovascular disease; METS, metabolic equivalents, WHR, waist-to-hip ratio.

**Supplementary Table 4. Associations of obesity categories with incident cardiovascular diseases and mortality outcomes**

|  | **WHR category** | **BMI category** | | **Combined BMI – WHR category** | | | | |
| --- | --- | --- | --- | --- | --- | --- | --- | --- |
| **Incident outcome** | **Elevated WHR** | **Overweight (BMI 25- 29.9)** | **Obese  (BMI > 30)** | **Normal BMI - elevated WHR** | **Overweight - normal WHR** | **Overweight - elevated WHR** | **Obese - normal WHR** | **Obese - elevated WHR** |
| Any CVD | 1.27* | 1.16* | 1.53* | 1.22* | 1.13* | 1.30* | 1.51* | 1.68* |
|  | [1.25, 1.30] | [1.13, 1.18] | [1.49, 1.57] | [1.17, 1.26] | [1.10, 1.17] | [1.26, 1.34] | [1.45, 1.58] | [1.63, 1.73] |
|  | 1.58x10^-119^ | 1.30x10^-35^ | 1.09x10^-247^ | 1.45x10^-22^ | 1.72x10^-14^ | 5.32x10^-70^ | 4.42x10^-79^ | 8.66x10^-254^ |
| Ischaemic heart disease | 1.34* | 1.22* | 1.48* | 1.33* | 1.20* | 1.44* | 1.45* | 1.70* |
|  | [1.30, 1.37] | [1.18, 1.25] | [1.44, 1.53] | [1.27, 1.40] | [1.15, 1.26] | [1.39, 1.50] | [1.36, 1.53] | [1.64, 1.77] |
|  | 2.09x10^-103^ | 2.72x10^-39^ | 3.28x10^-126^ | 4.00x10^-29^ | 1.99x10^-17^ | 9.83x10^-80^ | 4.94x10-35 | 6.72x10^-157^ |
| Atrial fibrillation | 1.18* | 1.09* | 1.58* | 1.04 | 1.06* | 1.13* | 1.61* | 1.61* |
|  | [1.14, 1.21] | [1.06, 1.13] | [1.53, 1.64] | [0.99, 1.10] | [1.02, 1.11] | [1.09, 1.18] | [1.52, 1.70] | [1.55, 1.68] |
|  | 2.18x10^-29^ | 3.25x10^-8^ | 1.61x10^-155^ | 0.1494 | 0.0082 | 5.29x10^-10^ | 9.80x10^-58^ | 5.89x10^-118^ |
| Heart failure | 1.36* | 1.10* | 1.74* | 1.24* | 1.04 | 1.26* | 1.71* | 1.94* |
|  | [1.30, 1.42] | [1.04, 1.15] | [1.66, 1.83] | [1.14, 1.34] | [0.97, 1.12] | [1.19, 1.34] | [1.57, 1.87] | [1.83, 2.06] |
|  | 5.75x10^-48^ | 1.71x10^-4^ | 2.17x10^-109^ | 1.93x10^-7^ | 0.2557 | 3.37x10^-14^ | 1.82x10^-35^ | 4.14x10^-103^ |
| All-cause mortality | 1.18* | 0.91* | 1.07* | 1.21* | 0.87* | 1.04 | 1.01 | 1.19* |
|  | [1.15, 1.21] | [0.89, 0.94] | [1.04, 1.10] | [1.15, 1.26] | [0.84, 0.91] | [1.00, 1.08] | [0.95, 1.06] | [1.14, 1.23] |
|  | 2.24x10^-36^ | 3.72x10^-11^ | 1.53x10^-5^ | 1.24x10^-16^ | 1.26x10^-11^ | 0.0273 | 0.8099 | 1.99x10^-20^ |
| CVD mortality | 1.46* | 1.09 | 1.47* | 1.47* | 1.06 | 1.39* | 1.38* | 1.82* |
|  | [1.35, 1.59] | [0.99, 1.19] | [1.34, 1.61] | [1.27, 1.70] | [0.91, 1.23] | [1.23, 1.57] | [1.12, 1.69] | [1.62, 2.06] |
|  | 6.43x10^-19^ | 0.0649 | 5.72x10^-16^ | 4.29x10^-7^ | 0.4596 | 7.66x10^-8^ | 0.0020 | 3.67x10^-22^ |

**Supplementary Table 4 footnote.** Results are hazard ratios, 95% confidence intervals and p-values from Cox proportional hazards regression in the full data set. Each cell represents one model. Hazard ratios reflect the increased hazard associated with category status, compared to normal obesity (normal WHR, normal BMI respectively). All models are adjusted by age, sex, ethnicity, deprivation, smoking, alcohol consumption frequency, processed meat intake, education, physical activity, diabetes, hypertension and high cholesterol.

Abbreviations: CVD, cardiovascular disease, BMI, body mass index; METS, metabolic equivalents, WHR waits-to-hip ratio

**Supplementary Table 5: CMR metrics by obesity category**

| CMR | Imaging set | Normal BMI - normal WHR | Normal BMI - elevated WHR | Overweight - normal WHR | Overweight - elevated WHR | Obese - normal WHR | Obese - elevated WHR |
| --- | --- | --- | --- | --- | --- | --- | --- |
| N | 32,107 | 9,432 | 3,383 | 5,446 | 7,931 | 1,466 | 4,449 |
| LVEDV (ml) | 148.2 (33.8) | 139.4 (31.1) | 145.8 (33.8) | 145.5 (33.3) | 155.0 (33.7) | 146.8 (29.4) | 160.7 (35.2) |
| LVEDV index (ml/m^2^) | 79.1 (14.1) | 80.7 (13.9) | 80.2 (15.0) | 78.7 (14.0) | 79.3 (14.2) | 75.1 (12.1) | 76.7 (13.8) |
| LVM (g) | 86.6 (22.4) | 76.0 (18.5) | 84.4 (19.2) | 82.4 (20.7) | 94.8 (20.7) | 83.4 (18.6) | 102.3 (24.2) |
| LVM index (g/m^2^) | 46.0 (8.7) | 43.9 (8.2) | 46.4 (8.2) | 44.5 (8.6) | 48.4 (8.5) | 42.6 (7.6) | 48.7 (9.2) |
| LVM: LVEDV | 0.59 (0.09) | 0.55 (0.07) | 0.58 (0.08) | 0.57 (0.08) | 0.62 (0.09) | 0.57 (0.08) | 0.64 (0.10) |
| LVEF (%) | 59.5 (6.1) | 60.0 (5.8) | 59.2 (6.2) | 59.9 (5.9) | 58.8 (6.4) | 60.5 (5.9) | 59.1 (6.5) |
| LVGFI (%) | 47.5 (6.9) | 49.4 (6.7) | 47.3 (6.7) | 48.5 (6.6) | 45.7 (6.7) | 49.0 (6.6) | 45.3 (6.8) |
| T1 mapping (ms) | 932 (36) | 942 (35) | 931 (34) | 932 (35) | 922 (35) | 935 (37) | 926 (36) |
| LAV (ml) | 73.0 (23.3) | 67.3 (20.3) | 67.3 (22.2) | 72.8 (21.4) | 75.2 (23.5) | 79.2 (22.0) | 83.6 (26.8) |
| LAV index (ml/m^2^) | 39.1 (11.3) | 39.1 (11.2) | 37.1 (11.6) | 39.5 (10.7) | 38.6 (11.4) | 40.6 (10.4) | 40.0 (12.1) |
| LAEF (%) | 61.3 (9.1) | 61.8 (8.5) | 62.0 (9.2) | 61.9 (8.6) | 61.0 (9.6) | 60.9 (8.6) | 59.3 (10.1) |
| PDA AoD(10^-3^/mmHg) | 2.42 (1.14) | 2.57 (1.27) | 2.35 (1.13) | 2.48 (1.19) | 2.29 (0.99) | 2.46 (1.12) | 2.28 (0.96) |
| ASI (m/s) | 9.56 (2.69) | 9.02 (2.69) | 9.72 (2.74) | 9.36 (2.64) | 10.05 (2.67) | 9.33 (2.57) | 10.06 (2.52) |

**Supplementary Table 5 footnote:** Continuous data are given in mean (standard deviation).

Abbreviations: index, indexation to body surface area; LVEDV, left ventricular end-diastolic volume; LVM, left ventricular mass; LVM:LVEDV, concentricity index; LVEF, left ventricular ejection fraction; LVGFI, left ventricular global function index, LAV, left atrial volume, LAEF, left atrial ejection fraction, PDA AoD, aortic distensibility at the proximal descending aorta; ASI, arterial stiffness index

**Supplementary Table 6. Associations between CMR metrics and incident outcomes**

|  |  | **Model 1** |  | **Model 2** |  | **Model 3** |  |
| --- | --- | --- | --- | --- | --- | --- | --- |
| **Exposure** | **Outcome** | **HR, 95% CI** | **p-value** | **HR, 95% CI** | **p-value** | **HR, 95% CI** | **p-value** |
| LVEDV | Any CVD | 1.32* [1.23, 1.42] | 9.13x10-14 | 1.34* [1.25, 1.44] | 2.15x10-15 | 1.32* [1.23, 1.42] | 2.14x10-14 |
|  | Ischaemic heart disease | 1.11 [1.01, 1.22] | 0.0367 | 1.14* [1.04, 1.26] | 0.0071 | 1.13 [1.03, 1.24] | 0.0119 |
|  | Atrial fibrillation | 1.51* [1.39, 1.63] | 1.91x10-24 | 1.52* [1.41, 1.65] | 1.76x10-26 | 1.50* [1.39, 1.62] | 3.52x10-24 |
|  | Heart failure | 1.93* [1.76, 2.11] | 4.73x10-44 | 1.94* [1.77, 2.13] | 9.08x10-45 | 1.89* [1.72, 2.08] | 1.00x10-40 |
|  | All-cause mortality | 1.15 [1.03, 1.29] | 0.0144 | 1.17* [1.05, 1.31] | 0.0048 | 1.17* [1.05, 1.31] | 0.0047 |
|  | CVD mortality | 1.74* [1.43, 2.12] | 3.92x10-8 | 1.74* [1.43, 2.12] | 2.52x10-8 | 1.69* [1.39, 2.06] | 1.07x10-7 |
| LVM | Any CVD | 1.68* [1.57, 1.80] | 1.70x10-48 | 1.69* [1.58, 1.81] | 7.49x10-50 | 1.60* [1.49, 1.72] | 6.75x10-38 |
|  | Ischaemic heart disease | 1.55* [1.42, 1.70] | 2.16x10-21 | 1.57* [1.43, 1.72] | 2.07x10-22 | 1.47* [1.34, 1.61] | 1.74x10-15 |
|  | Atrial fibrillation | 1.71* [1.57, 1.86] | 7.76x10-37 | 1.72* [1.59, 1.88] | 2.27x10-37 | 1.67* [1.53, 1.82] | 2.29x10-30 |
|  | Heart failure | 2.26* [2.03, 2.50] | 7.15x10-54 | 2.27* [2.05, 2.52] | 2.42x10-54 | 2.14* [1.92, 2.38] | 1.40x10-42 |
|  | All-cause mortality | 1.28* [1.14, 1.45] | 3.85x10-5 | 1.29* [1.14, 1.45] | 2.99x10-5 | 1.28* [1.13, 1.45] | 7.31x10-5 |
|  | CVD mortality | 1.90* [1.51, 2.38] | 4.09x10-8 | 1.92* [1.53, 2.42] | 2.79x10-8 | 1.86* [1.46, 2.37] | 5.18x10-7 |
| LVM: LVEDV | Any CVD | 1.26* [1.19, 1.34] | 2.75x10-15 | 1.25* [1.18, 1.33] | 4.69x10-14 | 1.19* [1.12, 1.27] | 1.51x10-8 |
|  | Ischaemic heart disease | 1.35* [1.26, 1.45] | 6.59x10-17 | 1.33* [1.24, 1.42] | 7.06x10-15 | 1.25* [1.16, 1.35] | 2.62x10-9 |
|  | Atrial fibrillation | 1.14* [1.05, 1.24] | 0.0013 | 1.13* [1.04, 1.23] | 0.0037 | 1.09 [1.00, 1.18] | 0.0605 |
|  | Heart failure | 1.14 [1.01, 1.29] | 0.0382 | 1.12 [0.99, 1.27] | 0.0669 | 1.04 [0.91, 1.18] | 0.5919 |
|  | All-cause mortality | 1.12 [1.02, 1.23] | 0.0160 | 1.10 [1.00, 1.21] | 0.0461 | 1.08 [0.99, 1.19] | 0.0962 |
|  | CVD mortality | 1.13 [0.87, 1.47] | 0.3711 | 1.12 [0.86, 1.46] | 0.4101 | 1.07 [0.81, 1.40] | 0.6323 |
| LVEF | Any CVD | 0.83* [0.78, 0.89] | 2.07x10-8 | 0.83* [0.78, 0.89] | 1.51x10-8 | 0.83* [0.77, 0.88] | 3.24x10-9 |
|  | Ischaemic heart disease | 0.90 [0.83, 0.98] | 0.0109 | 0.90* [0.83, 0.97] | 0.0085 | 0.89* [0.82, 0.96] | 0.004 |
|  | Atrial fibrillation | 0.78* [0.72, 0.85] | 4.68x10-9 | 0.79* [0.72, 0.85] | 3.83x10-9 | 0.79* [0.73, 0.85] | 4.09x10-9 |
|  | Heart failure | 0.52* [0.47, 0.57] | 2.29x10-47 | 0.52* [0.48, 0.57] | 2.46x10-46 | 0.54* [0.49, 0.58] | 3.90x10-44 |
|  | All-cause mortality | 0.84* [0.77, 0.93] | 3.54x10-4 | 0.84* [0.77, 0.93] | 3.21x10-4 | 0.85* [0.77, 0.93] | 3.66x10-4 |
|  | CVD mortality | 0.58* [0.47, 0.70] | 5.36x10-8 | 0.58* [0.48, 0.71] | 6.97x10-8 | 0.59* [0.48, 0.72] | 1.41x10-7 |
| LVGFI | Any CVD | 0.74* [0.69, 0.80] | 6.60x10-17 | 0.75* [0.69, 0.80] | 1.81x10-16 | 0.76* [0.71, 0.82] | 3.54x10-14 |
|  | Ischaemic heart disease | 0.76* [0.70, 0.83] | 7.88x10-10 | 0.77* [0.70, 0.84] | 2.33x10-9 | 0.79* [0.72, 0.86] | 9.52x10-8 |
|  | Atrial fibrillation | 0.74* [0.68, 0.81] | 2.58x10-10 | 0.75* [0.68, 0.82] | 5.07x10-10 | 0.76* [0.69, 0.84] | 8.24x10-9 |
|  | Heart failure | 0.44* [0.39, 0.50] | 3.11x10-41 | 0.45* [0.40, 0.51] | 3.84x10-40 | 0.47* [0.42, 0.53] | 8.12x10-36 |
|  | All-cause mortality | 0.80* [0.72, 0.88] | 1.57x10-5 | 0.80* [0.72, 0.89] | 3.23x10-5 | 0.81* [0.73, 0.90] | 6.72x10-5 |
|  | CVD mortality | 0.49* [0.38, 0.63] | 4.63x10-8 | 0.49* [0.38, 0.64] | 6.79x10-8 | 0.50* [0.38, 0.65] | 2.31x10-7 |
| Native T1 | Any CVD | 1.09 [1.01, 1.16] | 0.0199 | 1.07 [1.00, 1.15] | 0.0425 | 1.08 [1.01, 1.16] | 0.0206 |
|  | Ischaemic heart disease | 0.96 [0.88, 1.05] | 0.4293 | 0.95 [0.87, 1.04] | 0.2374 | 0.96 [0.88, 1.05] | 0.3437 |
|  | Atrial fibrillation | 1.21* [1.11, 1.33] | 3.92x10-5 | 1.20* [1.09, 1.31] | 1.01x10-4 | 1.21* [1.10, 1.32] | 5.57x10-5 |
|  | Heart failure | 1.42* [1.26, 1.61] | 1.35x10-8 | 1.39* [1.23, 1.57] | 1.20x10-7 | 1.40* [1.24, 1.58] | 3.94x10-8 |
|  | All-cause mortality | 1.25* [1.13, 1.38] | 1.02x10-5 | 1.23* [1.12, 1.36] | 4.06x10-5 | 1.23* [1.11, 1.36] | 4.54x10-5 |
| Native T1 | CVD mortality | 1.52* [1.17, 1.98] | 0.002 | 1.51* [1.16, 1.96] | 0.0021 | 1.51* [1.17, 1.96] | 0.0018 |
| LAV (log) | Any CVD | 1.40* [1.30, 1.50] | 2.27x10-20 | 1.41* [1.32, 1.52] | 2.16x10-21 | 1.37* [1.28, 1.47] | 2.93x10-18 |
|  | Ischaemic heart disease | 1.14* [1.04, 1.24] | 0.0031 | 1.15* [1.06, 1.25] | 0.0012 | 1.11 [1.02, 1.21] | 0.0127 |
|  | Atrial fibrillation | 1.92* [1.74, 2.12] | 7.88x10-39 | 1.94* [1.75, 2.13] | 8.57x10-40 | 1.87* [1.70, 2.07] | 5.67x10-36 |
|  | Heart failure | 1.74* [1.52, 1.98] | 1.46x10-16 | 1.75* [1.53, 1.99] | 6.34x10-17 | 1.65* [1.45, 1.88] | 8.57x10-14 |
|  | All-cause mortality | 1.11 [1.00, 1.23] | 0.0425 | 1.12* [1.01, 1.24] | 0.0296 | 1.11 [1.00, 1.23] | 0.0498 |
|  | CVD mortality | 1.57* [1.16, 2.13] | 0.0035 | 1.56* [1.15, 2.12] | 0.004 | 1.50* [1.11, 2.04] | 0.0091 |
| LAEF | Any CVD | 0.62* [0.58, 0.66] | 2.20x10-44 | 0.63* [0.59, 0.67] | 1.09x10-43 | 0.64* [0.60, 0.68] | 3.77x10-40 |
|  | Ischaemic heart disease | 0.81* [0.75, 0.87] | 7.88x10-8 | 0.81* [0.75, 0.88] | 1.45x10-7 | 0.83* [0.77, 0.90] | 2.83x10-6 |
|  | Atrial fibrillation | 0.47* [0.44, 0.51] | 2.30x10-79 | 0.48* [0.44, 0.52] | 1.18x10-77 | 0.49* [0.45, 0.53] | 1.26x10-71 |
|  | Heart failure | 0.57* [0.52, 0.63] | 1.38x10-31 | 0.58* [0.53, 0.63] | 2.94x10-30 | 0.60* [0.55, 0.66] | 8.11x10-26 |
|  | All-cause mortality | 0.88* [0.80, 0.96] | 0.0061 | 0.88* [0.81, 0.97] | 0.0092 | 0.89 [0.81, 0.98] | 0.0172 |
|  | CVD mortality | 0.66* [0.52, 0.83] | 4.79x10-4 | 0.66* [0.52, 0.84] | 6.06x10-4 | 0.67* [0.53, 0.86] | 0.0012 |
| PDA AoD (log) | Any CVD | 0.83* [0.76, 0.91] | 5.19x10-5 | 0.84* [0.77, 0.92] | 1.03x10-4 | 0.88* [0.80, 0.96] | 0.0039 |
|  | Ischaemic heart disease | 0.81* [0.72, 0.90] | 1.43x10-4 | 0.81* [0.73, 0.91] | 2.42x10-4 | 0.85* [0.76, 0.95] | 0.0057 |
|  | Atrial fibrillation | 0.95 [0.84, 1.08] | 0.4487 | 0.97 [0.85, 1.09] | 0.5848 | 1.00 [0.88, 1.14] | 0.9467 |
|  | Heart failure | 0.88 [0.73, 1.05] | 0.1506 | 0.89 [0.74, 1.06] | 0.1889 | 0.94 [0.78, 1.13] | 0.4907 |
|  | All-cause mortality | 0.93 [0.81, 1.07] | 0.3317 | 0.94 [0.82, 1.08] | 0.3573 | 0.94 [0.82, 1.08] | 0.3899 |
|  | CVD mortality | 0.74 [0.50, 1.09] | 0.1262 | 0.74 [0.50, 1.09] | 0.1286 | 0.77 [0.52, 1.14] | 0.1943 |
| ASI (imaging) | Any CVD | 1.08 [1.00, 1.15] | 0.0364 | 1.07 [1.00, 1.15] | 0.0479 | 1.06 [0.99, 1.14] | 0.0974 |
|  | Ischaemic heart disease | 1.07 [0.98, 1.17] | 0.1277 | 1.06 [0.97, 1.16] | 0.1647 | 1.05 [0.96, 1.15] | 0.2772 |
|  | Atrial fibrillation | 0.99 [0.90, 1.09] | 0.8338 | 0.98 [0.90, 1.08] | 0.7523 | 0.98 [0.89, 1.07] | 0.6392 |
|  | Heart failure | 0.92 [0.80, 1.05] | 0.2249 | 0.91 [0.80, 1.05] | 0.1877 | 0.90 [0.79, 1.03] | 0.1348 |
|  | All-cause mortality | 1.03 [0.93, 1.14] | 0.5857 | 1.02 [0.92, 1.14] | 0.6725 | 1.02 [0.92, 1.13] | 0.7195 |
|  | CVD mortality | 1.08 [0.79, 1.47] | 0.6341 | 1.08 [0.80, 1.48] | 0.6120 | 1.07 [0.79, 1.46] | 0.6465 |

**Supplementary Table 6 footnote.** Hazard ratios associated with 1 SD increase in CMR measures, 95% confidence intervals and p-values from Cox proportional hazards models relating LVEDV, LVM, LVM:LVEDV, LVEF, LVGFI, Native T1, LAEF, PDA AoD (log) and ASI to incident disease/events in the imaging subset. Each cell represents a different model. Model 1 = adjusted by age and sex, Model 2 = adjusted by Model 1 variables plus ethnicity, Townsend deprivation score, education, physical activity, alcohol consumption frequency, smoking and processed meat intake. Model 3 = adjusted by Model 2 variables plus diabetes, hypertension and high cholesterol.

Abbreviations: LVEDV, left ventricular end-diastolic volume; LVM, left ventricular mass; LVM:LVEDV, concentricity index; LVEF, left ventricular ejection fraction; LVGFI, left ventricular global function index, LAV, left atrial volume, LAEF, left atrial ejection fraction, PDA AoD, aortic distensibility at the proximal descending aorta; ASI, arterial stiffness index

**Supplementary Table 7. Mediation results for BMI-outcome associations**

| **Outcome** | **CMR metric** | **Total effect** | **Direct effect of obesity** | **Obesity via CMR** | **Obesity via Diabetes** | **Obesity via High cholesterol** | **Obesity via Hypertension** |
| --- | --- | --- | --- | --- | --- | --- | --- |
| **Ischaemic** | LVEDV | 0.155* (100%) | 0.002 ( 1%) | 0.040* (26%) | 0.017* (11%) | 0.025* (17%) | 0.070* (45%) |
| **heart disease** | LVM | 0.225* (100%) |  | 0.132* (59%) | 0.014 ( 6%) | 0.025* (11%) | 0.054* (24%) |
|  | LVM: LVEDV | 0.177* (100%) |  | 0.083* (47%) | 0.012* ( 7%) | 0.022* (12%) | 0.059* (34%) |
|  | LVEF | 0.125* (100%) | 0.008 ( 5%) | 0.004* ( 3%) | 0.016* (13%) | 0.024* (19%) | 0.074* (60%) |
|  | LVGFI | 0.157* (100%) | 0.001 ( 1%) | 0.049* (32%) | 0.013* ( 8%) | 0.025* (16%) | 0.068* (43%) |
|  | Native T1 | 0.130* (100%) | 0.002 ( 1%) | 0.011* ( 9%) | 0.017* (13%) | 0.023* (18%) | 0.076* (59%) |
|  | LAV (log) | 0.141* (100%) | 0.004 ( 3%) | 0.028* (19%) | 0.018* (13%) | 0.023* (17%) | 0.068* (49%) |
|  | LAEF | 0.127* (100%) | 0.004 ( 3%) | 0.015* (12%) | 0.016* (13%) | 0.023* (18%) | 0.068* (54%) |
|  | PDA AoD (log) | 0.115* (100%) | 0.005 ( 3%) | 0.009* ( 8%) | 0.019* (16%) | 0.023* (20%) | 0.060* (52%) |
|  | ASI (imaging) | 0.119* (100%) | 0.004 ( 3%) | 0.009 ( 7%) | 0.018* (15%) | 0.021* (18%) | 0.068* (57%) |
| **Atrial** | LVEDV | 0.151* (100%) | 0.001 (0%) | 0.079* (53%) | 0.015* (10%) | 0.001 (1%) | 0.055* (37%) |
| **fibrillation** | LVM | 0.206* (100%) |  | 0.153* (74%) | 0.012 (6%) |  | 0.041* (20%) |
|  | LVM: LVEDV | 0.135* (100%) | 0.029 (16%) | 0.037* (29%) | 0.013 (11%) | -0.002 (-1%) | 0.058* (46%) |
|  | LVEF | 0.113* (100%) | 0.030 (18%) | 0.006* (6%) | 0.014 (13%) |  | 0.064* (63%) |
|  | LVGFI | 0.130* (100%) | 0.009 (5%) | 0.052* (41%) | 0.011 (9%) |  | 0.058* (46%) |
|  | Native T1 | 0.131 (100%) | 0.073 (47%) | -0.018 (-16%) | 0.013* (12%) | -0.004 (-3%) | 0.066* (59%) |
|  | LAV (log) | 0.224* (100%) |  | 0.153* (68%) | 0.015* (7%) |  | 0.056* (25%) |
|  | LAEF | 0.123* (100%) | 0.003 (2%) | 0.049* (41%) | 0.010 (8%) |  | 0.060* (49%) |
|  | PDA AoD (log) | 0.153* (100%) | 0.078 (43%) | 0.003 (2%) | 0.015* (11%) |  | 0.057* (43%) |
|  | ASI (imaging) | 0.100* (100%) | 0.021 (14%) |  | 0.015 (15%) |  | 0.065* (71%) |
| **Heart failure** | LVEDV | 0.236* (100%) |  | 0.112* (48%) | 0.024* (10%) | 0.009 (4%) | 0.092* (39%) |
|  | LVM | 0.298* (100%) |  | 0.200* (67%) | 0.019* (6%) | 0.008 (3%) | 0.072* (24%) |
|  | LVM: LVEDV | 0.163* (100%) | 0.009 (4%) | 0.017 (10%) | 0.022* (13%) | 0.010 (7%) | 0.105* (66%) |
|  | LVEF | 0.147* (100%) | 0.005 (3%) | 0.016* (11%) | 0.016 (11%) | 0.010 (7%) | 0.100* (69%) |
|  | LVGFI | 0.231* (100%) | 0.002 (1%) | 0.120* (52%) | 0.013 (6%) | 0.008 (3%) | 0.088* (38%) |
|  | Native T1 | 0.127* (100%) | 0.020 (11%) | -0.042* (-36%) | 0.017* (15%) | 0.011 (9%) | 0.120* (101%) |
|  | LAV (log) | 0.255* (100%) |  | 0.123* (48%) | 0.019* (8%) | 0.013 (5%) | 0.099* (39%) |
|  | LAEF | 0.166* (100%) |  | 0.044* (27%) | 0.014 (8%) | 0.012 (7%) | 0.095* (57%) |
|  | PDA AoD (log) | 0.145* (100%) |  | 0.011 (9%) | 0.017 (11%) | 0.011 (8%) | 0.106* (72%) |
|  | ASI (imaging) | 0.158* (100%) | 0.008 (4%) |  | 0.019 (12%) | 0.016* (10%) | 0.115* (74%) |

**Supplementary Table 7 footnote.** BMI: body mass index. Average effect and proportion mediated (effect / total effect) for multiple mediation models between BMI (exposure) and incident events in the imaging subset. Mediated via diabetes, hypertension and high cholesterol plus one cardiovascular metric at a time. Each row represents one model. All models are adjusted by age, sex, smoking, alcohol intake frequency, ethnicity, Townsend deprivation score, education, physical activity and processed meat intake. Mediation analyses were conducted with the mmabig package in R, with 400 bootstrapped samples. An asterisk indicates an effect where the semiparametric confidence interval does not contain zero.

**Supplementary Table 8. Mediation summary for WHR-outcome associations**

| **Outcome** | **CMR metric** | **Total effect** | **Direct effect of obesity** | **Obesity via CMR** | **Obesity via Diabetes** | **Obesity via High chol** | **Obesity via Hypertension** |
| --- | --- | --- | --- | --- | --- | --- | --- |
| **Ischaemic** | LVEDV | 0.269* (100%) | 0.101 (35%) | 0.054* (21%) | 0.018* ( 7%) | 0.032* (13%) | 0.065* (25%) |
| **heart disease** | LVM | 0.300* (100%) | 0.004 ( 1%) | 0.191* (64%) | 0.018* ( 6%) | 0.032* (11%) | 0.055* (19%) |
|  | LVM: LVEDV | 0.269* (100%) | 0.057* (19%) | 0.113* (43%) | 0.014 ( 5%) | 0.028* (11%) | 0.057* (22%) |
|  | LVEF | 0.270* (100%) | 0.132* (47%) | 0.024* ( 9%) | 0.016* ( 6%) | 0.031* (12%) | 0.067* (26%) |
|  | LVGFI | 0.279* (100%) | 0.073 (24%) | 0.096* (35%) | 0.014* ( 5%) | 0.031* (11%) | 0.065* (24%) |
|  | Native T1 | 0.244* (100%) | 0.126* (49%) |  | 0.018* ( 7%) | 0.030* (13%) | 0.071* (30%) |
|  | LAV (log) | 0.278* (100%) | 0.150* (52%) | 0.019* ( 7%) | 0.017* ( 6%) | 0.030* (11%) | 0.062* (23%) |
|  | LAEF | 0.274* (100%) | 0.150* (53%) | 0.016* ( 6%) | 0.016* ( 6%) | 0.030* (12%) | 0.062* (24%) |
|  | PDA AoD (log) | 0.259* (100%) | 0.143* (52%) | 0.014* ( 6%) | 0.019* ( 8%) | 0.029* (12%) | 0.054* (22%) |
|  | ASI (imaging) | 0.266* (100%) | 0.148* (54%) | 0.012 ( 5%) | 0.018* ( 7%) | 0.026* (10%) | 0.062* (24%) |
| **Atrial fibrillation** | LVEDV | 0.200* (100%) | 0.002 (1%) | 0.124* (62%) | 0.018* (9%) |  | 0.055* (28%) |
|  | LVM | 0.277* (100%) |  | 0.219* (79%) | 0.014 (5%) |  | 0.044* (16%) |
|  | LVM: LVEDV | 0.155* (100%) | 0.035 (17%) | 0.044* (30%) | 0.016 (11%) |  | 0.060* (42%) |
|  | LVEF | 0.148* (100%) | 0.024 (12%) | 0.042* (30%) | 0.015 (11%) |  | 0.066* (47%) |
|  | LVGFI | 0.181* (100%) | 0.007 (3%) | 0.100* (56%) | 0.013 (7%) |  | 0.060* (34%) |
|  | Native T1 | 0.137* (100%) | 0.078* (48%) | -0.022 (-24%) | 0.016 (14%) | -0.003 (-2%) | 0.068* (64%) |
|  | LAV (log) | 0.181* (100%) | 0.002 (1%) | 0.106* (59%) | 0.018 (10%) |  | 0.055* (30%) |
|  | LAEF | 0.138* (100%) | 0.016 (9%) | 0.049* (37%) | 0.010 (7%) | 0.001 (1%) | 0.061* (46%) |
|  | PDA AoD (log) | 0.155* (100%) | 0.069 (37%) | 0.006 (4%) | 0.017 (13%) | 0.002 (1%) | 0.061* (45%) |
|  | ASI (imaging) | 0.118* (100%) | 0.035 (23%) |  | 0.016 (15%) |  | 0.066* (63%) |
| **Heart failure** | LVEDV | 0.312* (100%) |  | 0.181* (58%) | 0.027* (9%) | 0.012 (4%) | 0.092* (30%) |
|  | LVM | 0.400* (100%) |  | 0.293* (73%) | 0.022 (5%) | 0.009 (2%) | 0.077* (20%) |
|  | LVM: LVEDV | 0.169* (100%) | 0.007 (3%) | 0.016 (9%) | 0.026* (16%) | 0.012 (7%) | 0.108* (66%) |
|  | LVEF | 0.238* (100%) |  | 0.104* (44%) | 0.019 (8%) | 0.011 (5%) | 0.103* (43%) |
|  | LVGFI | 0.362* (100%) |  | 0.245* (68%) | 0.017 (5%) | 0.007 (2%) | 0.092* (25%) |
|  | Native T1 | 0.104* (100%) | 0.014 (10%) | -0.066* (-77%) | 0.022 (22%) | 0.014 (14%) | 0.120* (131%) |
|  | LAV (log) | 0.231* (100%) | 0.003 (1%) | 0.089* (39%) | 0.023* (10%) | 0.015 (6%) | 0.102* (44%) |
|  | LAEF | 0.181* (100%) | 0.005 (2%) | 0.050* (29%) | 0.016 (9%) | 0.012 (6%) | 0.099* (55%) |
|  | PDA AoD (log) | 0.165* (100%) | 0.008 (3%) | 0.015 (10%) | 0.019 (11%) | 0.011 (7%) | 0.112* (69%) |
|  | ASI (imaging) | 0.168* (100%) | 0.011 (5%) |  | 0.021 (13%) | 0.021 (13%) | 0.115* (70%) |

**Supplementary Table 8 footnote:** WHR: waist-hip-ratio. Average effect and proportion mediated (effect / total effect) for multiple mediation models between waist-hip-ratio (exposure) and incident events in the imaging subset. Mediated via diabetes, hypertension and high cholesterol plus one cardiovascular metric at a time. Each row represents one model. All models are adjusted by age, sex, smoking, alcohol intake frequency, ethnicity, Townsend deprivation score, education, physical activity and processed meat intake. Mediation analyses were conducted with the mmabig package in R, with 400 bootstrapped samples. An asterisk indicates an effect where the semiparametric confidence interval does not contain zero.
